# Potential barriers to and facilitators of civil society organization engagement in increasing immunization coverage in Odukpani Local Government Area of Cross River State, Nigeria: an implementation research

**DOI:** 10.1186/s12961-021-00697-y

**Published:** 2021-08-11

**Authors:** Aniekan Etokidem, Festus Nkpoyen, Comfort Ekanem, Enagu Mpama, Anastasia Isika

**Affiliations:** 1 Department of Community Medicine, University of Calabar Teaching Hospital, Calabar, Cross River State Nigeria; 2 Department of Sociology, University of Calabar, Calabar, Cross River State Nigeria; 3 Department of Clinical Governance, Cross River State Ministry of Health, Calabar, Cross River State Nigeria

**Keywords:** Civil society organization (CSO), Engagement, Immunization, Coverage, Vaccine, Demand, Uptake, Facilitators, Barriers

## Abstract

**Background:**

Civil society organizations (CSOs) are important in health care delivery. They have the potential to play significant roles in immunization-related services, such as advocacy, health education, demand creation and resource mobilization. Their roles are often indispensable, diverse and beneficial in reducing infant morbidity and mortality due to vaccine-preventable diseases. This study explored the potential barriers to and facilitators of CSO engagement in increasing immunization coverage in Odukpani Local Government Area of Cross River State, Nigeria.

**Methods:**

The study adopted qualitative data collection methods. Twenty-two focus group discussion (FGD) sessions, three in-depth interviews (IDIs) and 26 key informant interviews (KIIs) were conducted. Appropriate guides (FGD guide, IDI guide and KII guide) were used to conduct face-to-face interviews and the discussions. The FGDs, KIIs and IDIs were audio-recorded and transcribed. A framework analysis approach involving five key stages of analysis (familiarization with data, identification of thematic framework, indexing, charting, mapping/interpretation) was used for data analyses and presentation.

**Results:**

CSOs encounter barriers in the course of their immunization advocacy, communication and social mobilization due to male child preference, leading to shielding of male children and not allowing them to be given immunization, as well as patriarchy, safety concerns, religious concerns, anti-vaccine misinformation and rumours, low perception of effectiveness and efficacy of vaccines, inaccessibility of localities, low health literacy and superstitious beliefs. Various community structures, such as the institution of the village head, elders’ council and town crier (announcer), and the existence of change agents, act as facilitators of immunization advocacy and uptake. Factors such as traditional control mechanisms including masquerades and religion act as either barriers or facilitators depending on the community and the mode of deployment. CSO members are willing to overcome these barriers and leverage the facilitators.

**Conclusions:**

For successful engagement in immunization-related services, there are barriers in the study area that CSOs should overcome, such as male child preference and geographic inaccessibility, as well as facilitators that they should leverage such as traditional information dissemination systems and enforcement of compliance by the chiefs and elders’ council.

## Background

Civil society organizations (CSOs) can be defined as non-market and non-state organizations outside of the family in which people organize themselves to pursue shared interests in the public domain [1]. CSOs have a long history of involvement in public health [2]. These organizations have worked in partnership with governments to extend health care services to marginalized communities, particularly in remote, hard-to-reach rural areas. As observed by Greer et al. [3], service delivery is perhaps the most common attraction of CSOs for policy-makers. CSOs play a role in health care delivery in areas that other major key players like the state may not be able or may be unwilling to undertake. CSOs, in partnership with government, play a key role in implementing immunization programmes. In many countries, they deliver up to 65% of immunization services, as well as strengthening health systems, training health workers and supporting logistics [4].

In Nigeria, health care and general living conditions are poor, especially for women and children, thus resulting in high infant, under-5 and maternal mortality ratios [5, 6]. CSO efforts are therefore necessary for ameliorating some of these conditions. In Cross River State of Nigeria, women and children under the age of 5, who are the most vulnerable, constitute 22% and 20% of the total population of the state, respectively [7]. According to the Cross River State Strategic Health Development Plan (2010–2015) [7], “*the decay in Primary Health Care (PHC) services delivery and the overall functioning of the health system in Cross River State has over the years led to the poor health status of people of the State”*. Health indicators such as maternal mortality ratio (2000/100 000) and under-5 (176/1000) and infant (120/1000) mortality rates rank Cross River State amongst the states with the highest maternal and child deaths in Nigeria [7]. The commonest causes of infant mortality in the state include preventable diseases such as malaria, measles, malnutrition, diarrhoea and pneumonia [7].

Immunization coverage in Cross River State is low, as only 42% of children aged 12–23 months are fully immunized [7]. Children in the state who do not receive routine immunization suffer this fate because of geographical inaccessibility, inadequate health manpower in rural areas, and sociocultural factors that fuel anti-vaccine sentiments, amongst others. Odukpani Local Government Area (LGA) has communities located in terrains which are extremely difficult for community health workers to access. Apart from the fact that access to some communities requires crossing large bodies of water, some cannot even be accessed at all during rainy seasons. Recurrent communal clashes between communities in the LGA and those of neighbouring Akwa Ibom State also render some of these communities unsafe for immunization service providers [8, 9].

Grassroots CSOs, being indigenous associations, have the capacity to penetrate hard-to-reach communities to carry out immunization-related activities such as advocacy and sensitization on the importance of immunization. The main objective of this study was to assess potential barriers to and facilitators of CSO engagement in increasing immunization coverage in Odukpani LGA of Cross River State, Nigeria. In addition, this study aimed to identify and map CSOs active in health generally and in immunization specifically in the LGA and to synthesize and document key learnings from the existing body of knowledge on CSO participation in immunization services delivery.

## Methods

### Study setting

The study took place in Odukpani, a rural LGA in Cross River State of Nigeria. Odukpani has an estimated population of 257,800 [10]. There are 13 political wards in the LGA. The LGA is characterized by hard-to-reach riverine areas.

### Research design

The study used a qualitative cross-sectional design.

### Sampling

Multistage sampling was used. In the first stage, 11 grassroots CSOs were purposively selected (Tables 1, 2). They were selected from 37 CSOs spread across the 13 political wards in the LGA. In the second stage, 10–12 members were purposively selected from each organization. To ensure that each group was homogeneous, the sex and sociodemographic characteristics of the participants were considered in the selection. The sociodemographic characteristics of the participants in the 11 FGD groups (total of 129 in all) are shown in Table 3. Using a similar procedure, 10 categories of community members who were not members of CSOs were purposively selected. Eight to 12 members were selected from each of these categories, making a total of 113 participants in the 10 FGD groups (Table 4). The selection of the CSO members and non-CSO community members was on the basis of previous involvement or willingness to be engaged in health or immunization-related services. The selection was done across the 13 political wards in the LGA.

**Table 1:** Details regarding data collection methods

Data collection method	Category of participants	Number of categories	Number of participants
Focus group discussion	CSO members	11	129
Focus group discussion	Non-CSO members	10	113
Focus group discussion	Ward Development Committee	1	12
Key informant interview	CSO leaders	26	26
In-depth interview	PHC coordinator (retired)	1	1
In-depth interview	PHC coordinator (incumbent)	1	1
In-depth interview	Religious leader	1	1

**Table 2:** CSO participants

S/N	Category of CSO participants	Number of participants
1	Role model mother networks	12
2	Market women’s association	12
3	Market men’s association	12
4	Female farmers’ association	12
5	Fishermen’s association	12
6	Community youth organization	12
7	Male church association	11
8	Female church association	12
9	Twins Foundation	10
10	Traditional birth attendants	12
11	Male farmers’ association	12
	Total	129

**Table 3:** Sociodemographic characteristics of CSO participants

Variables	Number of respondents (*n* = 129)	Percentage
Sex		
Male	66	51.16
Female	63	48.84
Age (in years)		
20–29	21	16.28
30–39	29	22.48
40–49	26	20.16
50–59	28	21.71
60+	25	19.37
Religion		
Christianity	120	93.02
Traditional	9	6.98
Marital status		
Single	27	20.93
Married	65	50.39
Separated	11	8.53
Divorced	5	4.27
Widowed	21	16.28
Educational attainment		
No formal education	9	6.98
Obtained primary education	32	24.80
Obtained secondary education	79	61.24
Obtained tertiary education	9	6.98

**Table 4:** Categories of non-CSO community members

S/N	Respondent category	Number of participants
1	Male community leaders	10
2	Male youth	12
3	Female community leaders	11
4	Elderly men	12
5	Elderly women	12
6	Pregnant women	12
7	Mothers of infants	12
8	Young women	12
9	Political leaders	8
10	Religious leaders	12
	Total	113

Leaders (the president or secretary) of 26 CSOs that were not involved in the FGDs were purposively selected for key informant interviews. Two CSOs were selected from each of the 13 political wards in the LGA, giving a total of 26 CSOs. A religious leader, (the Coordinator of the Christian Association of Nigeria, Odukpani Chapter), the immediate-past PHC coordinator and the incumbent PHC coordinator of the local government were also purposively selected for in-depth interviews (IDI). The IDIs and key informant interviews (KIIs) were added as triangulation strategies to make the findings of the study more robust.

### Data collection instruments and data collection

Qualitative data were obtained through focus group discussions (FGD), KIIs and IDIs using FGD, IDI and KII guides, respectively. The guides were developed and pretested by the team.

The FGD sessions sought to identify facilitators of and barriers to CSO engagement in immunization-related services. The sessions also aimed to elicit ways that community members and CSOs could work together in addressing the identified barriers and harnessing identified facilitators to increasing demand for and uptake of immunization services in the LGA. The KIIs and IDIs were conducted to obtain further insight into what the interviewees knew about barriers to and facilitators of demand for and uptake of immunization and how to overcome these barriers and leverage the facilitators.

Each FGD session had a moderator, a recorder and a note-taker. Recording was done using an electronic recorder and an iPad after seeking the consent of participants. Each FGD session involved eight to 12 participants, and a session lasted 60 to 90 minutes. Written informed consent was obtained from each participant.

### Data analysis

#### Categorization of participants

The data on categorization of participants according to the aspect of the study they participated in (FGD, KII or IDI) and their sociodemographic characteristics were analysed and are presented in Tables 1, 2, 3, 4, 5 and 6.

**Table 5:** Sociodemographic characteristics of non-CSO members

Variable	Number of respondents (*n* = 113)	Percentage
Sex		
Male	53	46.90
Female	60	53.10
Age (years)		
20–29	24	21.24
30–39	26	23.01
40–49	20	17.70
50–59	19	16.81
60+	24	21.24
Religion		
Christianity	109	96.46
Traditional	4	3.54
Marital status		
Single	22	19.47
Married	64	56.64
Separated	8	7.08
Divorced	3	2.65
Widowed	16	14.16
Educational status		
No formal education	8	7.08
Obtained primary education	43	38.05
Obtained secondary education	50	44.25
Obtained tertiary education	12	10.62

**Table 6:** Number of participants that mentioned specific issues according to data collection methods

Issue discussed	Number of participants that mentioned the issue		
	FGD	KII	IDI
Difficult terrain and geographic inaccessibility	72	26	3
Cultural festivities	13	18	
Patronage of traditional medical practice	60	26	3
Wrongful attribution of disease to immunization	38	7	
Effect of religious beliefs and practices, belief in traditional deities	18	24	1
Protection of the firstborn child			1
Patriarchy, male child preference, women’s low decision-making power and lack of male involvement	20	2	1
Myths, misconceptions, superstitions and misinformation regarding immunization	60	20	2
Distrust in vaccinators who are not from the community and the poor attitude of some female caregivers	13	12	
Health system challenges	31	17	2
Religion	38	24	3
Traditional social control mechanisms	130	26	2
Existence of pro-vaccine community members	3	2	1
Community perception of CSO engagement	15	24	
Assistance that CSOs expect from the community	18	26	
Past experience in immunization	32	26	
CSOs assist in repairing the road	2		
CSOs address myths and misconceptions regarding immunization	3	2	
CSOs navigate hard-to-reach areas and assist the vaccinators	7	13	1

### Qualitative data processing and analysis

A framework analysis approach involving five key stages of analysis was used: familiarization with the data; identification of a thematic framework; indexing; charting; and finally, mapping and interpretation [11]. In order to ensure trustworthiness and credibility of the data analysis, each stage was carried out by more than one research team member.

Searching through the data, several themes were identified, including CSO involvement in creating demand for and uptake of immunization; barriers that CSOs encounter; shielding of the first child (both male and female) from immunization; patriarchy, male child preference; and shielding of male children (irrespective of birth order) from immunization, amongst others.

Themes were organized into categories and subcategories. Data extracts on central themes were reported verbatim. Data mapping and interpretation were done guided by the charts. The number of participants that mentioned specific issues according to data collection methods is indicated in Table 6.

## Results

### Barriers to increasing demand for and uptake of immunization encountered by CSOs

Results of FGDs, KIIs and IDIs showed that CSOs encountered several barriers, as described below:

#### Geographic inaccessibility, lack of means of transportation and communal clashes

Geographic inaccessibility, lack of means of transportation and communal clashes were highlighted as barriers:

*We don’t have canoe to cross the water to another village, we don’t have a good road to pass to another village and even to the health centre. We trek there, sometimes machine (motorcycle) cannot enter.* (Male, 40, KII)

This was corroborated by a key informant:

*The challenge is that the road is very bad, not motorable. Some places you have to park your vehicle and trek up to 2–3 hours and get tired*. (Male, 63, KII)

Adding the communal clashes dimension, another key informant noted:

*There are no machines (motorcycles) to carry them due to bad roads. Sometimes also there are communal clashes in the riverine areas.* (Male, 51, KII)

#### Patronage of traditional medicines

There is a preference for traditional medicine and loathing of orthodox medicine, including immunization, as an interviewee pointed out:

*These people called native doctors, herbalists are problems, and I can say that most times due to improper orientation an herbalist will tell you that, “What is it that you are going there to do? I have the needed herbs required that can take care of these things.”* (Male, 45, IDI)

Regarding the faith that community members have in herbalists, one discussant said that:

*They believe in taking the children to herbalist to carry out incision on the children.* (Female, 30, FGD)

Some mothers believe in giving their children herbs, roots and enema instead of immunization, as narrated by a focus group discussant:

*Some believe in herbs and roots. Some believe in giving the baby enema. They have a belief that immunization will rather make their children sick.* (Male, 32, FGD)

#### Wrongful attribution of disease and death to immunization

Some community members believe that immunization can cause paralysis or even make children sick. One pregnant respondent stated:

*For some people after immunization the child will have paralysis so they don’t like immunizing their children.* (Female, 22, FGD)

Some community members even believe that immunization can make children become sick and die, as described by a young woman:

*Some of the reasons why some mothers refuse to take their children for immunization is because some nurses after administering the immunization, the children come back with high fever, some become sick and some die.* (Female, 24, FGD)

One mother is said to have refused to avail her child of immunization because she believed the first one died because of it, as discussed by a religious leader:

*Barriers exist in different levels like a mother that refused it because the first child died after immunization.* (Male, 65, FGD)

#### Shielding of firstborn children, both male and female

In some families, firstborn children (both male and female) must be shielded against anything that is perceived to be harmful, including immunization. This was described by an interviewee:

*There are families that will have some laws that in this family all our first children must not be immunized. Those things could stop or hinder mothers from accessing facilities for the services rendered.* (Female, 59, IDI)

#### Male child preference

Another participant gave an interesting dimension to the manifestation of male child preference whereby fathers allow female children to be immunized while shielding male children so that they would not die:

*Men, especially those who have one male child, can prevent the male child from receiving immunization and instead allow it to be given to the girls so that the male child would not die.* (Female, 60, FGD)

A discussant in another group said something similar:

*Some used to say please don’t take my male child for immunization so that he will not die.* (Female, 30, FGD)

#### Patriarchy, low economic status of women and low decision-making power

A traditional birth attendant recounted how these played out while trying to find out why a pregnant woman did not receive tetanus toxoid immunization.

*She said no, that since her pregnancy was four months, she had asked the husband to give her money to go for immunization. The husband told her, “I won’t give you even one Naira, even if that immunization is free you won’t go. My mother gave birth to all of us, she never went to the hospital. The moment you people are pregnant you look for a means of getting money from a man for your personal use.”* (Female, 47, FGD)

The opinion of another traditional birth attendant made this more succinct:

*Some of these pregnant women need this money to register in the hospital, a pregnant woman also needs to change from her normal dressing of tight clothes and high heel shoes, but when this is not provided for her by the husband, she feels so ashamed to come out and mingle with other women.* (Female, 60, FGD)

Throwing more light on the role of men in immunization decision-making and the low decision-making power of women, another FGD participant stated:

*Most husbands do not give their wives the authority to get their children immunized, even though the wives are willing.* (Male, 35, FGD)

#### Rumours regarding immunization

Immunization-related rumours are replete in the study area. One role model mother described how it was rumoured that “all” children who were immunized “somewhere” died:

*There was this rumour that children were immunized somewhere and they all died. Because of that a lot of people refused to immunize their children.* (Female, 39, FGD)

Another rumour even associated immunization with kidnapping of children, as related by a religious leader:

*We have heard many negative news or rumours concerning kidnapping of children by strangers in the pretext of giving them immunization.* (Male, 63, FGD)

#### Distrust in vaccinators who are not from the community

Caregivers sometimes refuse to have their children immunized because the health workers are not members of their communities. According to one CSO participant,

*If the workers come from outside Odukpani Local Government Area like from other states to work here, mothers will surely refuse it… if they see strange workers, none will trust them.* (Male, 35, FGD)

#### Health system challenges

A Ward Development Committee member complained that sometimes community members would go to the health facility for immunization and other services but would not find any health care provider. This discourages the community members. According to him:

*We do not like the idea that we have few hands in the health centre. Most times the health centre is closed and not opened to cater for community people.* (Female, 42, FGD)

A similar challenge was pointed out by a role model mother:

*There is no one to manage the health post, no nurse. The nurses can stay away for two to three months before they come to check on the place or open the health post. We need a functional health centre with staff.* (Female, 67, FGD)

### Facilitators of demand for and uptake of immunization services

The study also identified some facilitators of demand for and uptake of immunization as follows:

#### Existence of vaccine advocates in the communities

A vaccine advocate community member held the perception that immunization makes children beautiful and intelligent:

*You can see the children of these days so beautiful and intelligent because of immunization.* (Male, 66, FGD)

Another advocate, a key informant, highlighted the benefits of immunization:

*Nowadays, we have seen the benefits of immunization because before now, most people believe in taking roots and herbs. They will not like to take their children to the health centre, but because they have seen the benefits of immunization, people will be rushing out to take the immunization… children are no more dying because of that.* (Female, 45, KII)

#### Existence of several immunization information dissemination channels

##### Traditional information dissemination channels

All the communities have well-established traditional information dissemination systems which they use to disseminate information about upcoming immunization activities.

*In my community, we have town criers (town announcers) that can go around ringing bell and announcing immunization messages.* (Male, 35, KII)

##### Information dissemination through the school system

Schools play a key role in facilitating immunization uptake. Information about immunization is passed to school children, who in turn pass it to their parents and other community members.

*Immediately information about immunization gets to the teachers or the head of the school, they will pass the information to the pupils in schools.* (Male, 31, KII)

#### Existence of alternative immunization points

Schools and churches also serve as alternative immunization points, as pointed out by a focus group discussant:

*Sometimes the immunization is given in churches and schools in the communities.* (Male, 32, FGD)

#### Enforcement of compliance by traditional rulers and council of elders

Traditional rulers have a pivotal role to play in the uptake of immunization as mentioned during a FGD.

*After the chief has made or given his orders for all with children to bring them out to be immunized, the secretary of the CSO will take down names of community members who comply, and those who do not will be asked to pay fine.* (Male, 34, FGD)

The village council of elders also plays a key role in enforcing compliance with immunization uptake, according to a ward development committee member:

*Also, there used to be meeting of the village council of elders concerning the exercise. They sometimes keep an injunction and fine for those who default in bringing out their children to receive immunization.* (Female, 43, FGD)

### Factors that act both as facilitators and as barriers

The study identified certain factors that act as facilitators and as barriers, depending on the circumstance and the community. Such factors include:

#### Religion

One in-depth interviewee identified religion as a facilitator:

*The hospital takes care of the physical life of that person, so as a church, it is our place to continue to preach or announce that our people should run away from self-medication, thy should embrace free immunization.* (Male, 45, IDI)

This is in agreement with the opinion of a focus group discussant:

*Church pastors also encourage members to take part in immunization exercise.* (Male, 29, FGD)

On the contrary, a focus group discussant identified religion as a barrier because some churches do not believe in the efficacy of immunization and therefore do not allow their members to avail their children of these services.

*Some churches which deal on spirits, special oils and water make those members not to receive immunization. They used to say “in our church we don’t receive immunization, our pastor has handkerchief and special oil, our pastor can pray and we get healed when we are sick”. * (Male, 60, FGD)

Another focus group discussant corroborated this:

*I am a witness. During one of the Immunization Plus Days, one church did hinder members from receiving immunization until the officials were called from Abuja (the nation’s capital) before the church apologized and then agreed for the children to be immunized.* (Female, 35, FGD)

A key informant also said something similar:

*Some people, their religion also affects them, they don’t take drugs, injection or medicine.* (Male, 25, KII)

#### Community social control mechanisms

Some community social control mechanisms are used to enforce compliance with immunization in some communities, for instance, cultural groups which have a high degree of social control. One key informant said that:

*Our village head sometimes uses Ekpe culture masquerade to force people out of their farms or homes to participate in immunization exercise. As people would not like to pay fine…, they will be forced to come out.* (Male, 25, KII)

Corroborating this, a traditional birth attendant said that:

*There are some villages where they use Ekpe cultural masquerade to inform people about immunization. They have injunctions so because of these injunctions, women comply by taking their children for immunization.* (Female, 51, FGD)

In another community, the same Ekpe masquerade was used as a barrier to access to immunization services, according to another key informant:

*I can say that Ekpe culture masquerade can also be a barrier as people cannot go for immunization when they play it, and there is one man who stopped his wife from taking the children to immunization, such a man can block the woman.* (Male, 59, KII)

This was also the opinion of a youth focus group discussant.

*Yes, some affect uptake of immunization services here such as cultural plays like Ekpe and Obon cultural masquerades.* (Male, 30, FGD)

### The role of CSOs in overcoming barriers and leveraging facilitators for increased immunization demand and uptake

#### Community perception of CSO engagement

The interviews showed that CSOs have good rapport with community members, and this builds trust and facilitates immunization service provision.

According to a key informant:

*Immunization is better now because most CSO members have good rapport with non-CSO community members and are close to them and the people trust and believe in them. So CSO participation in immunization programme is beneficial.* (Male, 27, KII)

#### Assistance that CSOs expect from community members

The CSOs expect some assistance from the communities in order to facilitate their immunization advocacy activities.

The CSOs expect community members to:

*…assist the group with means of transportation to move around and to carry other health personnel.* (Male, 23, FGD)

#### Assistance that CSOs expect from the government

In order to overcome identified barriers and leverage the facilitators, CSOs expect some assistance from the government.

*When government gives to us stipends as appreciation for the assistance, it motivates us, even though it is not salary.* (Male, 60, KII)

In the opinion of another key informant, the government’s assistance with means of transportation can facilitate the work of CSOs.

*Government should also give us transportation in form of speed boat or canoe to be able to reach riverine areas.* (Male, 32, KII)

#### Leveraging experience in community mobilization

Some CSO members already have experience in community mobilization for immunization, as recounted by one key informant:

*My experience on immunization programme is when we go out to give immunization, the house to house immunization we will go to a compound, we will knock, if there is any person in the compound, the person will come out, when we see the mark on the wall and know that there are children in the house.* (Female, 55, KII)

Another key informant elaborated on this role:

*I have been administering the drugs, the immunization drugs, the one they drop in the mouth, the polio vaccine. I also use marker. I mark the child on the hand, the left hand; immediately after that I also use marker to mark on the wall to show that this house has partaken in the immunization programme.* (Male, 43, KII)

#### CSOs assist in repairing the road

The study area is characterized by a poor road network with many potholes due to erosion. CSOs assist in repairing the roads, as averred by a member:

*During rainy season, we used to try to mend or repair the road according to our ability at least to make it a little accessible or manageable.* (Female, 35, FGD)

#### CSOs address myths and misconceptions regarding immunization

A CSO member narrated their role in disabusing people’s mind regarding immunization.

*Our association do go into the community to disabuse people’s mind of their misconceptions about immunization, that it does not kill children or give them problem but help prevent common diseases.* (Male, 32, FGD)

#### CSOs can navigate hard-to-reach areas

CSO members use their personal means of transportation to access hard-to-reach areas.

*Our association has two motorcycles, we have boats we will use boat to enter place that are not motorable or machine (motorcycle) cannot go.* (Male, 25, KII)

Another CSO does something similar in the riverine areas, as thus described by its president:

*At times we have to find engine boat to be able to take them (immunization workers) across the river to be able to visit the villages.* (Male, 60, KII)

For another CSO, they not only navigated interior areas, but they also helped with interpretation of immunization messages to the people:

*We have motorcycles to use and go to interior areas, we also have people who can interpret the immunization messages better for community members to understand.* (Male, 57, KII)

## Discussion

The study found that CSOs in the study community encounter barriers in the course of their immunization demand creation, such as geographic inaccessibility and lack of trust by community members. In a related study, Catholic Relief Services (CRS) documented that CSOs in Madagascar, Pakistan and Nigeria encountered similar barriers in their immunization initiatives [12]. Lack of trust in CSOs is not peculiar to community members, as there are instances where health workers also have a poor perception of them. The resultant lack of trust acts as a barrier to the immunization advocacy, communication and social mobilization activities of the CSOs [12]. Inadequate knowledge regarding immunization among community members is another major barrier identified by this study. The Civil Society in Malaria Control, Immunization and Nutrition (ACOMIN) had earlier identified this barrier in the communities in which it worked in Nigeria [13].

Patriarchy, which was identified as a barrier to immunization in this study, can take different forms. Firstly, it deprives women of decision-making ability and freedom of movement. In Pakistan, CSOs working on immunization found that women were often not allowed to leave home without a male chaperone. In Guinea, community change champions found that women were afraid that by putting an ink mark on the nails of their children, their husbands would find out that the children had been immunized, and this could lead to reprisal [12]. According to Makama, tradition or culture and religious beliefs in Nigeria, as a typical patriarchal society, see the wife as property [14]. The husband therefore feels capable and empowered to take decisions for and on behalf of his “property” even in matters concerning his “property’s” health and that of her children. This study found that sometimes the head of the family*,* that is the man, may not give the caregiver the permission to avail the child of immunization services*.* This problem posed by patriarchy is also found in other developing countries [15, 16]. Patriarchy might manifest in the form of male child preference. Male child preference ensures the perpetuity of patriarchy. Such preference may manifest in male children being more welcome to the family than females. This study found that fathers wanted to shield their male children from being vaccinated so that they would not die, while female children were presented for vaccination. The findings of an earlier study in Cross River State confirmed the pre-eminence given to male children [17].

Health manpower shortage was identified as a barrier. According to WHO, the Nigerian health sector is facing a major human resource crisis, with maldistribution of the available workforce and increasing brain drain resulting in shortage of critically needed health professionals [18]. Worse still, there is the challenge of low retention of health workers in rural areas due to lack of incentives [19].

Some factors act as both barriers and facilitators. In some communities, the Ekpe and Obon culture masquerades serve as barriers, as nonmembers, including women, should not be seen outside during the festivities, while in other communities these masquerades serve as facilitators, as they are used to enforce compliance with immunization uptake. Religion was another factor that served as a facilitator in some circumstances and as a barrier in others. While some religious leaders encouraged immunization and even allowed the exercise to be carried out inside their churches, other religious leaders would chase vaccinators away. Religion has always been a major factor in Nigeria’s immunization programme. In 2003, allegations by religious leaders that polio vaccination was meant to reduce the population of their adherents and that it also contained the human immunodeficiency virus led to the boycott of the programme in Northern Nigeria [20].

The study identified a number of roles that CSOs can play in overcoming identified barriers and leveraging identified facilitators towards increasing immunization demand and uptake in the LGA. CSOs in the study area were able to navigate hard-to-reach areas, just as was done by CSOs in Madagascar in the Reach Every Child immunization programme and in Pakistan, both leading to an increase in immunization coverage [12]. CSOs should explore partnerships with the government. The Global Alliance for Vaccines and Immunization (GAVI) has documented that with such partnership, CSOs in some countries have delivered up to 65% of immunization services and also contributed towards health system strengthening [21].

The CSOs can leverage existing community social mechanisms and institutions. For instance, the village heads and council of elders are highly revered in Nigerian communities. Their pro-immunization messages and instructions to community members would facilitate demand creation and uptake. The use of town criers (recently named “town announcers”) has been the method of conveying information to community members in Nigeria for decades [22, 23]. This is a useful method for disseminating to community members the immunization-related messages that CSOs and communities have harnessed. The use of cultural masquerades as a facilitator of immunization demand and uptake in some communities could be adopted throughout the LGA. With advocacy, the CSOs can get the buy-in of the traditional institutions across the LGA to get this done.

There are pro-vaccine advocates in some of the communities. One discussant attributed the beauty and intelligence of children in his community to immunization. Although there is no scientific evidence that immunization makes children “beautiful and intelligent”, the positive attitude shown by these community members portrays the fact that they could be used in immunization advocacy. In the course of their immunization advocacy activities, such persons could be persuaded to parade their healthy “beautiful and intelligent” children and make them part of the campaign, as evidence to community members that immunization makes a positive impact on children’s lives.

CSOs could overcome the resistance by religious leaders by emulating the Civil Society Human and Institutional Development Programme (CHIP) in Pakistan, which held a series of sensitization meetings with religious leaders until they eventually became immunization advocates [12].

As a means of tackling the challenge of health manpower shortage, CSO members could volunteer to be trained as village health workers (VHWs) who could assist the few health workers available in these rural communities. In Northern Nigeria as well as Zimbabwe, VHWs were found to be useful in community mobilization for, and a concomitant increase in, immunization coverage [24, 25].

## Limitations of the study

This study was conducted in only one of 18 LGAs in Cross River State. The findings may therefore not be generalizable to grassroots-based CSOs in other LGAs in the state.

## Conclusion

Grassroots CSOs in the study area have the capacity, capability and willingness to mobilize their communities towards increased demand for and uptake of immunization services. However, to be able to do this, CSOs must overcome several barriers which the study identified. They should also leverage on some facilitators which were also identified. The work of the CSOs can be further enhanced by collaboration with non-CSO community volunteers and the government, all working in partnership to bring immunization services to people in this rural community. This study has highlighted the facilitating role that CSOs can play in rural communities in increasing demand for and uptake of immunization services in a developing country with health manpower shortage and sociocultural, geographic, political and economic barriers.

## Data Availability

The data sets used and/or analyzed during the current study are available from the corresponding author on reasonable request.

## Abbreviations

CSO: Civil society organization
FGD: Focus group discussion
IDI: In-depth interview
KII: Key informant interview
LGA: Local Government Area
WHO: World Health Organization
VHW: Village health worker
